# Epstein-Barr Nuclear Antigen 1 Contributes to Nasopharyngeal Carcinoma through Disruption of PML Nuclear Bodies

**DOI:** 10.1371/journal.ppat.1000170

**Published:** 2008-10-03

**Authors:** Nirojini Sivachandran, Feroz Sarkari, Lori Frappier

**Affiliations:** Department of Molecular Genetics, University of Toronto, Toronto, Ontario, Canada; University of Southern California School of Medicine, United States of America

## Abstract

Latent Epstein-Barr virus (EBV) infection is strongly associated with several cancers, including nasopharyngeal carcinoma (NPC), a tumor that is endemic in several parts of the world. We have investigated the molecular basis for how EBV latent infection promotes the development of NPC. We show that the viral EBNA1 protein, previously known to be required to maintain the EBV episomes, also causes the disruption of the cellular PML (promyelocytic leukemia) nuclear bodies (or ND10s). This disruption occurs both in the context of a native latent infection and when exogenously expressed in EBV-negative NPC cells and involves loss of the PML proteins. We also show that EBNA1 is partially localized to PML nuclear bodies in NPC cells and interacts with a specific PML isoform. PML disruption by EBNA1 requires binding to the cellular ubiquitin specific protease, USP7 or HAUSP, but is independent of p53. We further observed that p53 activation, DNA repair and apoptosis, all of which depend on PML nuclear bodies, were impaired by EBNA1 expression and that cells expressing EBNA1 were more likely to survive after induction of DNA damage. The results point to an important role for EBNA1 in the development of NPC, in which EBNA1-mediated disruption of PML nuclear bodies promotes the survival of cells with DNA damage.

## Author Summary

Epstein-Barr virus (EBV) infects most people worldwide and is associated with several types of cancer due to its ability to induce cell proliferation. Only one viral protein, EBNA1, is expressed in all forms of EBV-associated tumors. Here, we have investigated whether EBNA1 directly contributes to the development of nasopharyngeal carcinoma (NPC), the most common EBV-associated tumor. We found that EBNA1 disrupts structures in the cell nucleus, called PML bodies, that are known to inhibit malignant transformation and to be important for cells to repair DNA that has been damaged due to exposure to carcinogenic agents. We show that EBNA1 interacts with and degrades the principal component of PML bodies. As a result, cells expressing EBNA1 are less able to repair their DNA and more likely to survive with DNA damage that could result in malignant transformation.

## Introduction

Epstein-Barr virus (EBV) is widely recognized as a causative agent of nasopharyngeal carcinoma (NPC), as most NPC tumors are monoclonal proliferations of latently EBV-infected cells [1]. Latent EBV infection in NPC involves expression of four viral proteins; two latent membrane proteins (LMP1 and LMP2), one nuclear protein (EBNA1) and one secreted protein (BARF1) [1],[2]. LMP1, LMP2 and BARF1 have all been reported to have cellular effects that may contribute to the development of NPC, although LMP1 and BARF1 are not consistently detected in all NPC tumors [3]–[5]. EBNA1 is required for the replication and stable persistence of EBV episomes in proliferating cells and is the only EBV protein that is expressed in all EBV-associated tumors [6]. EBNA1 enables the expression of the other EBV latency proteins, however, whether or not EBNA1 directly contributes to the development of tumors has not been clear.

A number of observations in the literature are consistent with a role for EBNA1 in the proliferation of EBV-positive cells. For example, interference with EBNA1 function in EBV-positive Burkitt's lymphoma cells, by overexpression of a dominant-negative EBNA1 mutant, increased cell death [7]. Similarly, down-regulation of EBNA1 in Raji Burkitt's lymphoma or EBV-positive epithelial cells by RNA interference decreased cell proliferation [8],[9]. However, since EBNA1 is needed to maintain the EBV episomes and to enhance expression of other latency proteins, it is not clear from the above observations whether EBNA1 is directly affecting cell proliferation or is functioning indirectly by enabling expression of other EBV gene products.

Other studies have investigated whether expressing EBNA1 in various EBV-negative cancer cells affects tumorgenicity. EBNA1 expression in HONE-1 NPC cells was found to increase primary tumor formation as well as metastases in nude mice [10]. EBNA1 expression in Hodgkin's lymphoma cells enhanced their ability to form tumors in non-obese diabetic-SCID mice but not in regular SCID mice [11]. In addition, Kaul et al [12] found that expression of EBNA1 in a breast carcinoma cell line promoted the rate of tumor growth in nude mice, reversed the growth inhibitory effect of the cellular Nm23-H1 protein and increased lung metastases.

The molecular basis for the observed effects of EBNA1 on cell proliferation are largely unknown, although an interaction between EBNA1 and the cellular ubiquitin specific protease, USP7 or HAUSP, has been proposed to be partially responsible [13]. USP7 binds and stabilizes p53 [14], and EBNA1 was found to block the USP7-p53 interaction *in vitro* by competing for the same binding pocket on USP7 [15]–[17]. In keeping with these findings, expression of EBNA1 (but not a USP7-binding mutant of EBNA1) in U2OS cells was shown to protect these cells from apoptosis in response to DNA damage by interfering with p53 stabilization [16]. However, USP7 is likely to have multiple cellular roles and the functional significance of the EBNA1-USP7 interaction remains to be determined in the context of latent EBV infection.

Few studies have investigated the role of EBNA1 in NPC. Studies on the contribution of EBV proteins to NPC in general have been hampered by the lack of EBV-positive NPC cell lines, since NPC cells tend to rapidly lose the EBV genomes when propagated in culture. The isolation of a NPC cell line (C666-1) that stably maintains EBV episomes [18] has greatly facilitated NPC studies, enabling a comparison to EBV-negative NPC cell lines. We have compared C666-1 cells to the EBV-negative NPC cell lines CNE2 [19] and HK1 [20] in order to better understand cellular alterations caused by EBV infection that may contribute to cell transformation. Here we show that EBV latent infection in NPC cells is associated with the disruption of host PML nuclear bodies (NBs) and that EBNA1 is entirely responsible for this effect. Consistent with the known importance of PML NBs in p53 activation and DNA damage responses, we also show that EBNA1 expression in NPC impairs both of these processes.

## Results

### EBNA1 disrupts PML NBs in NPC cells

**Figure 1:**
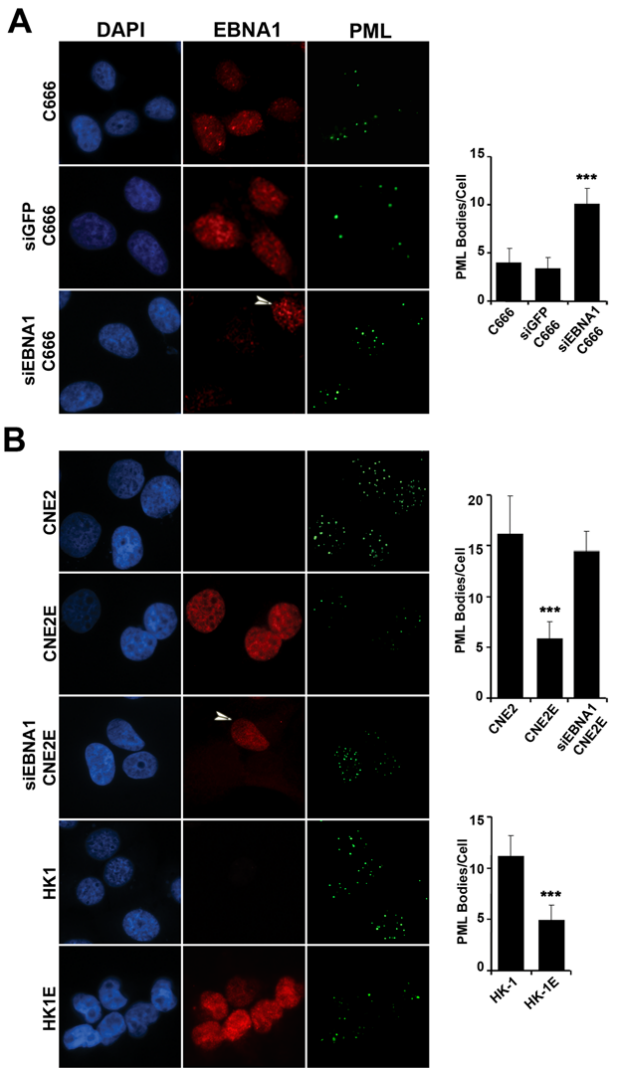
Immunofluorescence imaging of PML NBs in NPC cell lines. Log phase cells were fixed and stained for EBNA1 (red) and PML (green). The number of PML foci seen per cell was counted for 100 cells for each sample in three separate experiments and the average number with standard deviation is shown in the histograms, where *** denotes p values less than 0.0001 relative to the parental cell line. Exposure times of image capture were constant for all samples with the same antibody treatment. (A) EBV-positive C666-1 cells before and after treatment with siRNA against GFP (siGFP) or EBNA1 (siEBNA1) are shown. Arrowheads indicate a siEBNA1 treated cell that continued to express EBNA1 and can be used for comparison to neighboring silenced cells. (B) EBV-negative CNE2 and HK1 cell lines with (CNE2E, HK1E) and without stable EBNA1 expression are shown. CNE2E are also shown after silencing of EBNA1 expression where one of the three cells shown continues to express EBNA1 (arrowhead).

In initial studies comparing EBV-positive and EBV-negative NPC cell lines, we examined the host PML NBs, which have the PML protein as the main constituent and have been implicated in many important cellular processes [21]. We were particularly interested in examining PML NBs in the context of NPC because the disruption of PML NBs, or lack of the PML protein, is a factor in the development of several types of cancer [22],[23] and because DNA viruses are known to have mechanisms to disrupt PML NBs [24]. Immunofluorescence (IF) microscopy for PML revealed that C666-1 cells have considerably fewer PML NBs than do CNE2 or HK1 cells (average number per cell of 4, 16 and 11 respectively; Figure 1A and 1B), suggesting that some aspect of EBV infection disrupts PML NBs. We investigated whether this involved the EBNA1 protein by down-regulating EBNA1 expression with siRNA. This treatment greatly decreased EBNA1 expression in some but not all of the C666-1 cells allowing a direct comparison of silenced and non-silenced cells in the same culture (Figure 1A bottom row). The number of PML NBs was found to increase 2–3 fold upon EBNA1 silencing, as compared to non-treated cells or cells treated with siRNA against green fluorescence protein (GFP), indicating that EBNA1 contributes to PML disruption in C666-1.

**Figure 2:**
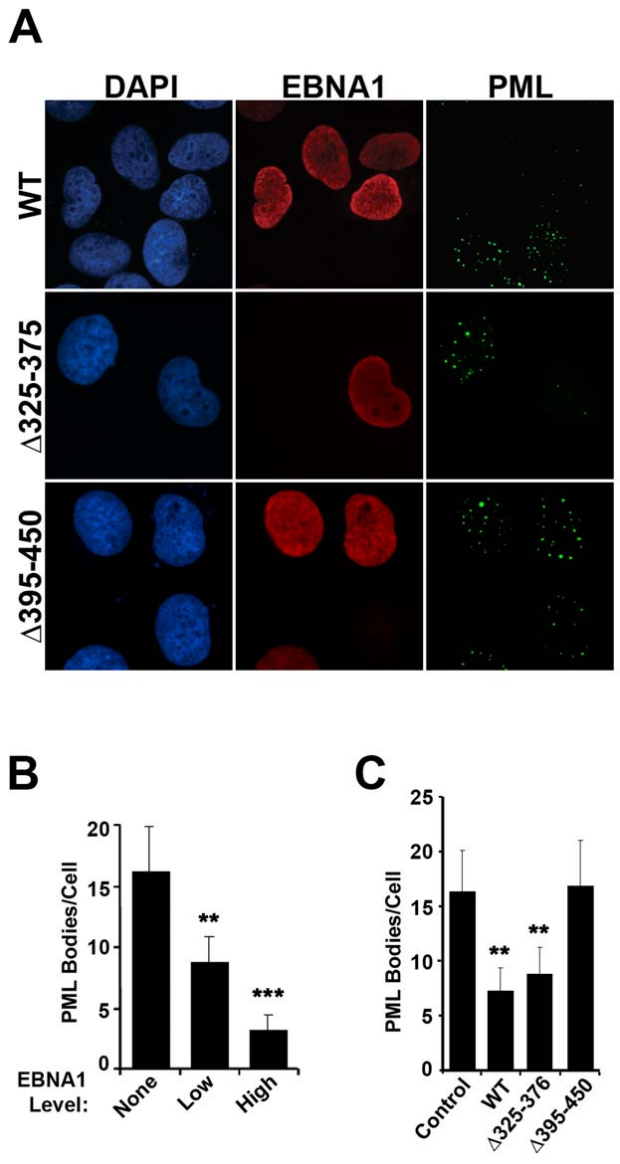
Transient expression of EBNA1 and EBNA1 mutants in CNE2 cells. (A) CNE2 cells were transiently transfected with a plasmid expressing EBNA1 or EBNA1 mutants Δ325–376 or Δ395–450, then stained for EBNA1 and PML. Both EBNA1-expressing and nonexpressing cells are shown 48 hrs post transfection. Exposure times of image capture were constant for all samples with the same antibody treatment. (B) Numbers of PML NBs per cell were counted 48 hours after expression of wildtype EBNA1. Cells were categorized into low and high EBNA1 expression depending on the intensity of EBNA1 staining. ** indicates 0.0001<p<0.001 and *** indicates p<0.0001 relative to untransfected cells. (C) The number of PML NBs were counted for all cells in (A) expressing wildtype or mutant EBNA1 proteins.

To determine whether EBNA1 expression was sufficient to disrupt PML NBs, we generated CNE2E and HK1E cell lines in which EBNA1 was constitutively expressed in CNE2 and HK1 NPC cells from an integrated cassette. EBNA1 expression levels in HK1E and CNE2E were shown by immunoblot to be approximately 2-fold and 3-fold higher than in C666-1, respectively (Figure S1). EBNA1 expression in both cell lines resulted in a notable decrease in the number of PML NBs per cell (Figure 1B). To further verify that this effect was caused by EBNA1, CNE2E cells were treated with siRNA to down-regulate EBNA1 expression. EBNA1 silencing restored the number of PML NBs to the level seen in the parent CNE2 cells (Figure 1B). We also examined whether transient expression of EBNA1 was sufficient to cause PML disruption. To this end, CNE2 cells were transfected with an EBNA1 expression plasmid and examined by IF 48 hrs later (Figure 2A). EBNA1 expression lowered the number of PML NBs in a dose-dependent manner, where PML NBs were decreased 5-fold in cells staining brightly for EBNA1 and decreased 3-fold in those with lighter EBNA1 staining (Figure 2B). Therefore EBNA1 expression has an immediate effect on PML NBs.

### EBNA1 lowers PML protein levels

**Figure 3:**
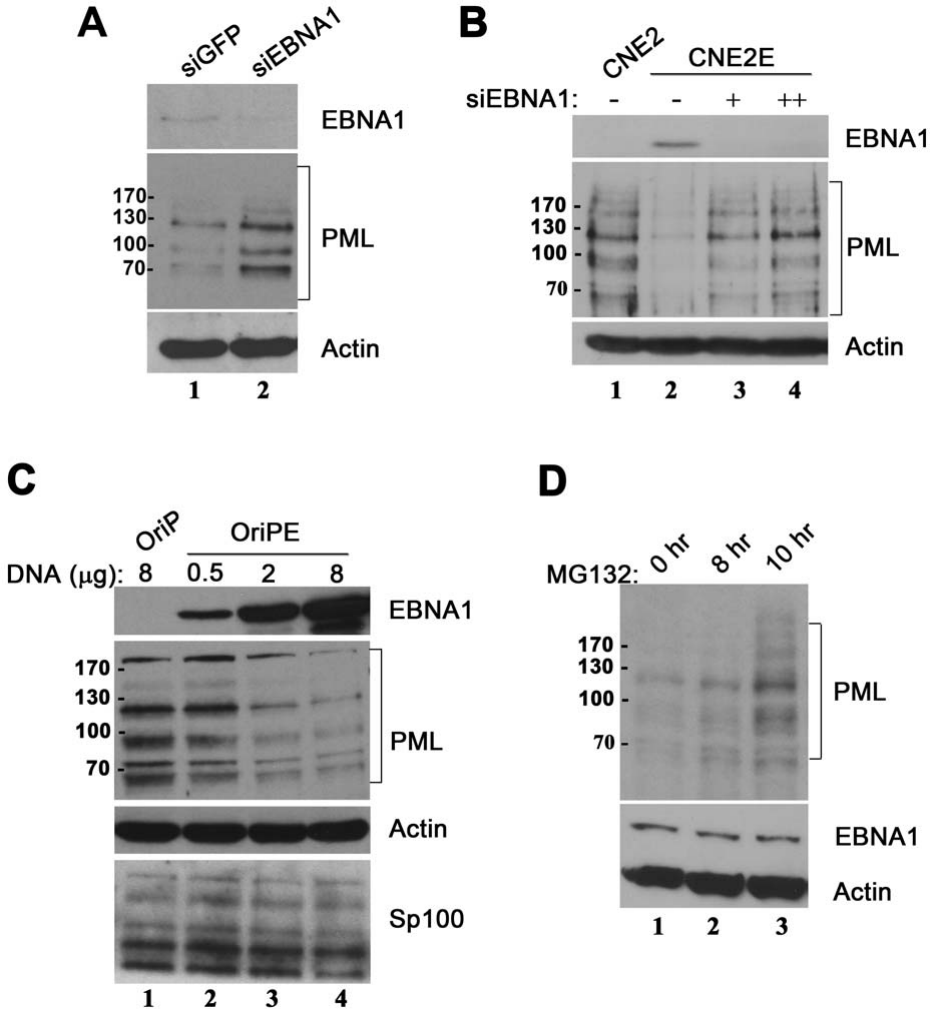
EBNA1 expression diminished PML protein levels. (A) C666-1 cells were treated with siRNA against EBNA1 (siEBNA1) or GFP (siGFP) then equal amounts of whole cell lysates were Western blotted and probed with an antibody recognizing all PML isoforms, EBNA1 and actin. (B) Equal amounts of whole cell lysates from CNE2 and CNE2E cells were Western blotted and probed as in A. Lysates from CNE2E cells after one (+) or two (++) rounds of transfection with siRNA against EBNA1 are also shown (lanes 3 and 4). (C) Lysates from CNE2 cells 48 hrs after transfection with the indicated amounts of an EBNA1 expression plasmid (OriPE) or the empty plasmid (OriP) were Western blotted for PML, EBNA1, actin or Sp100. (D) CNE2E cells were treated with MG132 proteasomal inhibitor for 0, 8 or 10 hours then equal amounts of lysates were blotted for PML, EBNA1 and actin.

Viral proteins have been found to decrease PML NBs either by inducing the degradation of the PML protein or by disrupting the interaction of PML proteins required to form NBs [24]. To address the mechanism by which EBNA1 expression disrupts PML NBs, we compared the levels of PML isoforms in CNE2 before and after stable (CNE2E) or transient expression of EBNA1 by Western blotting. PML is known to exist as several isoforms (comprised of alternative spliced and modified forms), resulting in multiple bands migrating between 60 and 200 Kda on PML immunoblots [25],[26]. Down-regulation of EBNA1 expression in C666 cells resulted in increased expression of all PML isoforms (Figure 3A). Similarly, EBNA1 expression in CNE2E resulted in a dramatic decrease in all PML isoforms (Figure 3B compare lanes 1 and 2), which was restored by silencing EBNA1 expression (Figure 3B lanes 3 and 4). In addition, transient EBNA1 expression in CNE2 decreased the level of all PML isoforms in a dose-dependent manner (Figure 3C). Therefore EBNA1 expression results in the loss of PML protein, as opposed to dispersal of PML from the foci. However the level of another PML NB component, Sp100, was unaffected by EBNA1 demonstrating the specificity of this effect (Figure 3C). Effects of EBNA1 expression on the level of PML mRNA was also examined by RT-PCR to rule out potential effects on PML transcription. As expected, no change in the level of PML transcripts was evident, indicating that the EBNA1-mediated PML effects were occurring at the protein level (Figure S2).

The EBNA1-induced loss of PML protein suggests that EBNA1 might be increasing the degradation of PML isoforms by the proteasome. We tested this possibility by examining the effect of blocking the proteasome in CNE2E cells with MG132 (Figure 3D). This treatment was found to increase the levels of all PML isoforms, and higher molecular weight forms suggestive of polyubiquitination also became visible. Therefore the loss of PML protein caused by EBNA1 is proteasome dependent.

### EBNA1 associates with PML NBs through a specific PML isoform

**Figure 4:**
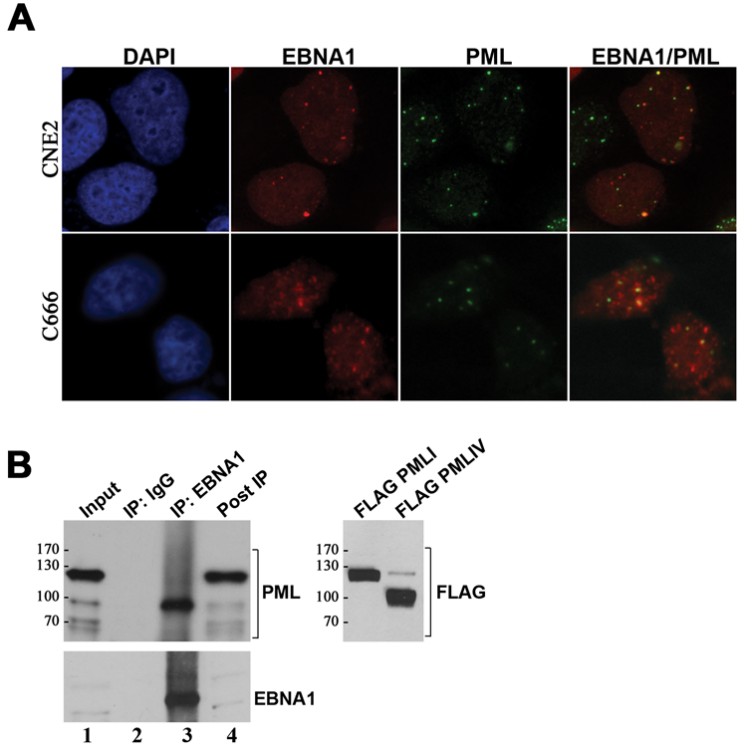
Interaction of EBNA1 with PML. (A) Immunofluorescence images of CNE2 cells transfected with pc3OriPE and of C666-1 cells are shown after staining for EBNA1 and PML. The transfected CNE2 cells shown are those expressing very low levels of EBNA1. (B) EBNA1 was immunoprecipitated from C666-1 cells with anti-EBNA1 antibody (IP:EBNA1). The starting lysate (Input) and protein remaining after IP (Post IP) are also shown, in each case representing 1/40^th^ of the lysate used in IP. The same lysate was also treated with IgG beads as a negative control (IP:IgG). All samples were Western blotted using antibodies against EBNA1 or all PML isoforms. In the right panel, the positions of FLAG-tagged PML isoform I (FLAG-PML I) and PML isoform IV (FLAG-PML IV) expressed in C666-1 cells are shown by Western blotting with anti-FLAG antibody.

In most cells, EBNA1 is found throughout the nucleus making it difficult to assess whether some of the EBNA1 localizes to PML NBs. However, some of the transiently transfected CNE2 cells expressed very low levels of EBNA1 and, in these cells, discreet EBNA1 foci were observed, many of which localized to PML NBs (Figure 4A). In addition, EBNA1 foci are frequently seen in C666-1, which naturally express low levels of EBNA1, and these foci often correspond to or overlap with PML NBs, even though few PML NBs are present in C666-1 (Figure 4A).

To further assess the interaction of EBNA1 with PML in the context of a latent infection, EBNA1 was immunoprecipitated from C666-1 and co-precipitating proteins were analysed for PML (Figure 4B). One PML isoform was consistently found to co-immunoprecipitate with EBNA1 (Figure 4B lane 3). Interestingly this did not correspond to the most prevalent PML band in the lysate (presumably isoforms I and II according to its size and abundance) but rather corresponded to a less abundant form consistent with the size of PML isoform IV [26]. EBNA1 was also found to preferentially bind PML IV over PML I when FLAG-tagged versions of these proteins were over-expressed in CNE2 cells along with EBNA1 (Figure S3).

### EBNA1-mediated disruption of PML NBs involves USP7

To gain insight into the mechanism by which EBNA1 induces loss of PML NBs and protein, we tested the ability of EBNA1 mutants to disrupt PML NBs after transfection in CNE2 cells (Figure 2A and 2C). Initially we tested the EBNA1 Δ325–376 mutant, as this mutation disrupts the interaction of EBNA1 with cellular chromatin and abrogates the transcriptional activation function of EBNA1 [27],[28]. However EBNA1 Δ325–376 disrupted PML NBs to the same degree as wildtype EBNA1 indicating that neither transcriptional activation nor strong chromatin interactions are required for the observed effects. An EBNA1 mutant, Δ395–450, that is fully functional for all of the known functions of EBNA1 (replication, segregation and transcriptional activation) but fails to bind the cellular USP7 protein was also tested for PML effects [13]. USP7 is known to be partially associated with PML NBs and associates with another herpesvirus protein (ICP0 or Vmw110 from herpes simplex virus) that also disrupts PML NBs through loss of PML protein [29]–[31]. Unlike wildtype EBNA1, Δ395–450 caused no obvious change in the number of PML NBs or the level of PML protein, suggesting that USP7 binding might be important for PML disruption.

**Figure 5:**
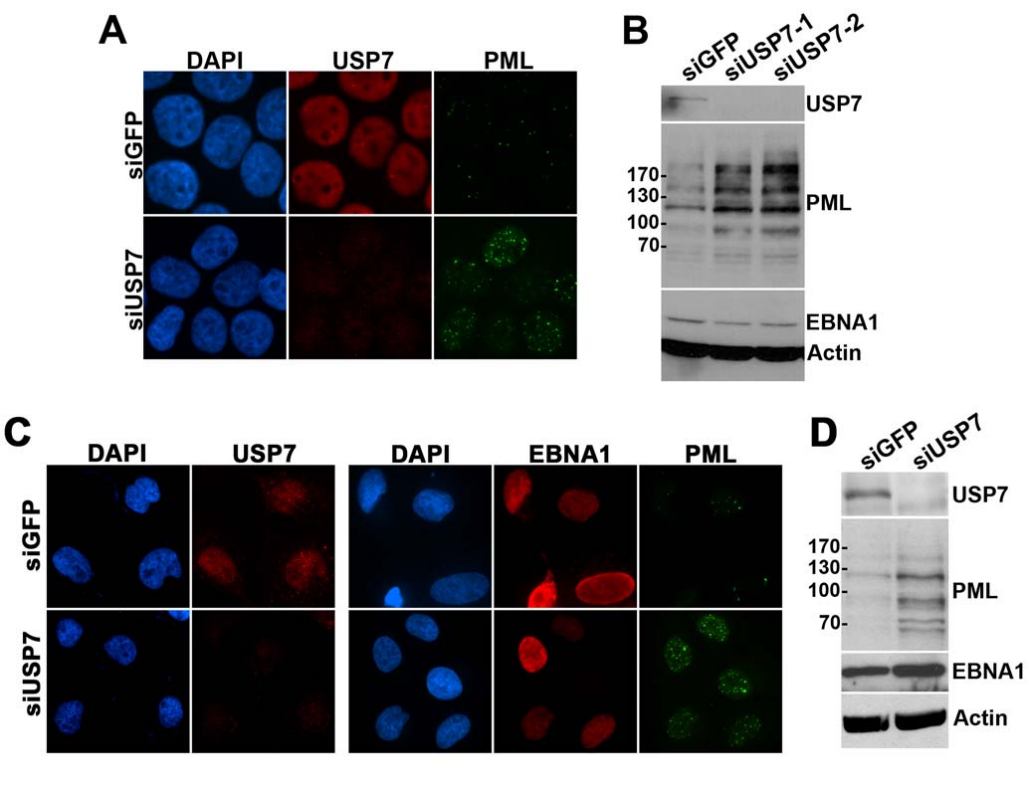
Effect of USP7 silencing on PML degradation by EBNA1. (A) CNE2E cells expressing EBNA1 were transfected with siRNA against USP7 or GFP (negative control) then stained for USP7 and PML. Exposure times of image capture were constant for all samples with the same antibody treatment. (B) Equal amounts of cell lysates from (A) were analysed by Western blotting with the indicated antibodies. siUSP7-1 and siUSP7-2 are duplicate samples treated with siRNA against USP7. (C) CNE2 cells were transfected with siRNA against GFP or USP7 (left panel) then were transfected with EBNA1 expression plasmid pc3OripE and stained for EBNA1 and PML (right panel). Exposure times of image capture were constant for all samples with the same antibody treatment. (D) Equal amounts of cell lysates from (C) were analysed by Western blotting after pretreatment with siGFP or siUSP7 followed by EBNA1 expression.

The role of USP7 in EBNA1-mediated disruption of PML NBs was further investigated by silencing USP7 by siRNA treatment in the CNE2E cells that are stably expressing EBNA1. USP7 silencing restored the number of PML NBs and the level of the PML protein (Figure 5A and 5B), indicating that EBNA1 does not disrupt PML NBs in the absence of USP7. Similar experiments were conducted in which CNE2 cells were transfected with siRNA against USP7 (or GFP as a negative control) prior to transient expression of EBNA1. The siUSP7 treatment was confirmed by IF to silence USP7 expression in virtually all of the CNE2 cells prior to transfection of the EBNA1 expression plasmid (Figure 5C, left panel). Cells pretreated with siUSP7 had numerous PML NBs regardless of whether or not EBNA1 was expressed, whereas EBNA1 continued to diminish PML NBs in cells pretreated with siGFP (Figure 5C, right panel). Similarly, pretreatment with siUSP7 but not siGFP interfered with EBNA1-induced loss of PML protein as determined by Western blotting (Figure 5D). Therefore EBNA1-mediated disruption of PML NBs requires USP7.

**Figure 6:**
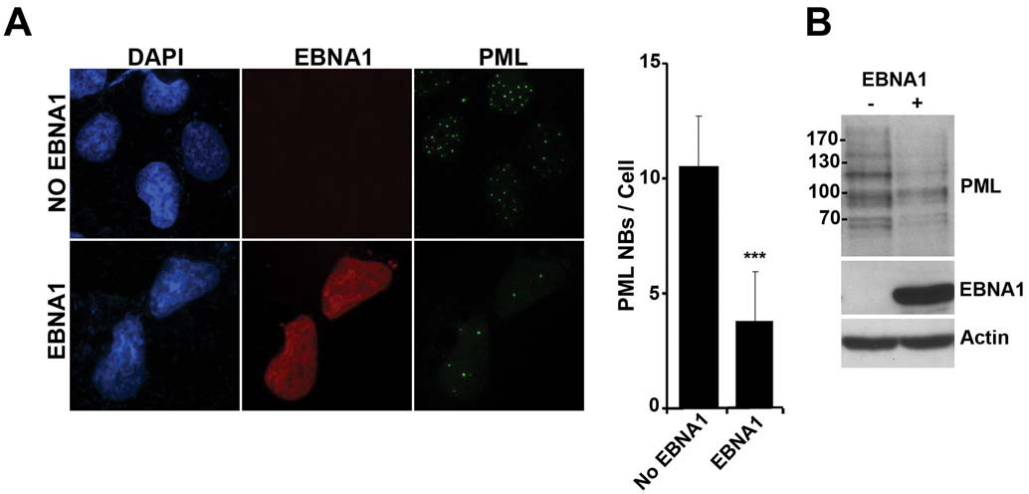
EBNA1-induced disruption of PML NBs in Saos-2 cells. p53-null Saos-2 cells were transiently transfected with the expression plasmid with or without the EBNA1 gene as in Figure 2. (A) Cells were stained for EBNA1, PML and DNA (DAPI) and visualized by fluorescence microscopy. The number of PML NBs per cell were counted and average numbers with standard deviations are shown in the histogram, where *** indicates p<0.0001. (B) Equal amounts of lysates from the transfected cells were analysed by Western blotting as in Figure 3.

Since USP7 can alter p53 levels [14],[32] and p53 induces PML transcription [33],[34], we wanted to ensure that the observed effects of EBNA1 on PML were not due to interference with p53 stabilization by USP7. Therefore we examined whether the EBNA1 effects on PML were independent of p53 by expressing EBNA1 in the p53-null Saos-2 cells. As shown in Figure 6, EBNA1 reduced the number of PML NBs per cell and the level of PML protein to a similar degree as in the NPC cells lines. Therefore the disruption of PML NBs by EBNA1 does not involve p53.

### EBNA1 interferes with p53 activation, DNA repair and apoptosis

**Figure 7:**
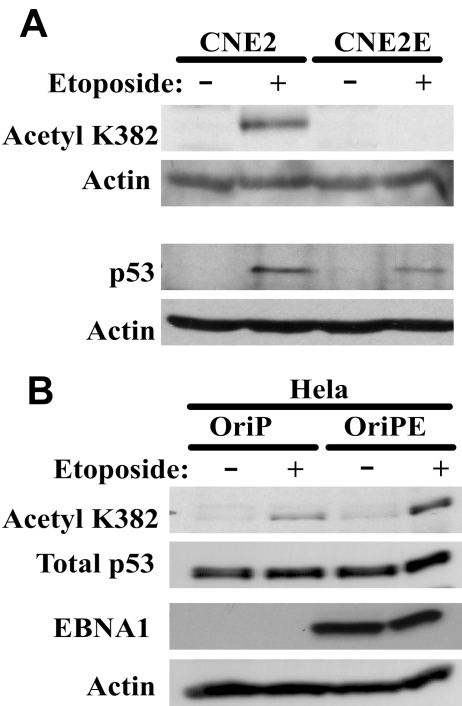
Effects of EBNA1 on p53 activation. (A) CNE2 and CNE2E cells were treated with etoposide (+) or left untreated (−) and equal amounts of total cell lysates were analysed by SDS-PAGE and Western blotting for p53 acetylated on K382 and total p53. Actin loading controls are also shown. (B) Hela cells were transfected with a plasmid lacking (oriP) or expressing (oriPE) EBNA1 then were treated with etoposide (+) or left untreated (−). Equal amounts of cell lysate were then analysed by Western blotting as in A.

Considerable data indicate that PML NBs are important for p53 activation, apoptosis and DNA repair which would have important consequences for the development of NPC. Therefore we asked whether the effect of EBNA1 on PML NBs was sufficient to disrupt these processes. PML NBs are required for the activation of p53 through acetylation [35],[36], and therefore we compared p53 activation in CNE2 and CNE2E cells after treatment with the DNA damaging agent etoposide (Figure 7A). We consistently observed that the EBNA1-expressing cells had an impaired ability to acetylate p53 (at K382) while the induction of p53 was affected to a lesser degree. In Hela cells, EBNA1 has no obvious effect on PML NBs (data not shown) and therefore we examined the effect of EBNA1 expression on p53 activation in Hela cells to verify that this effect involved PML NB disruption. As shown in Figure 7B, p53 acetylation in Hela cells occurred in response to etoposide treatment at least as efficiently in the presence of EBNA1 as in its absence. Therefore PML disruption by EBNA1 appears to be responsible for the lack of p53 acetylation.

**Figure 8:**
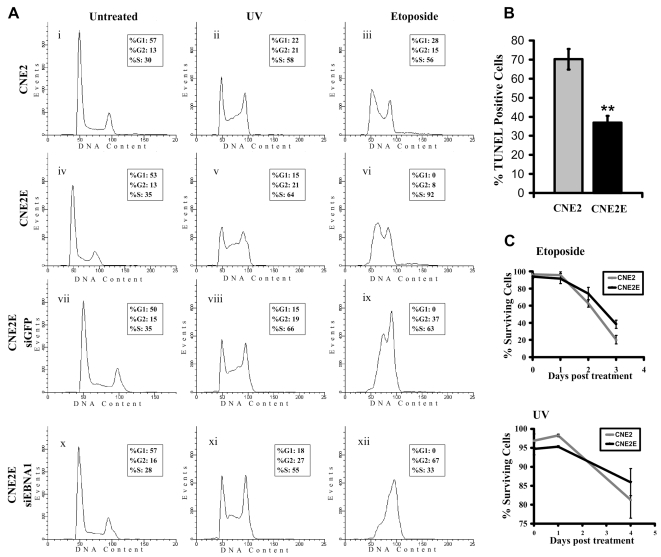
Effects of EBNA1 on DNA repair, apoptosis and cell survival. (A) CNE2 and CNE2E cells, before and after siRNA treatment for GFP (siGFP; negative control) or EBNA1 (siEBNA1), were treated with UV or etoposide or left untreated. 24 hrs later cells were fixed, stained with propidium idodide and analysed for DNA content by FACS. The percentage of cells in each cell cycle stage was determined using Modfit and is shown for each sample. (B) CNE2 and CNE2E cells were treated with etoposide then analysed by TUNEL assay. Average percentage of TUNEL-positive cells are shown from three experiments with standard deviation and 0.0001<p<0.001 (**). (C) CNE2 (grey) and CNE2E (black) cells were treated with etoposide (top graph) or UV (bottom graph) then grown for the indicated number of days. At each time point, cells were incubated with Trypan blue and the percentage of cells that excluded Trypan blue was determined. Experiments were performed in triplicate and average numbers with standard deviations are shown. The difference in cell survival with and without EBNA1 3 days post etoposide treatment is statistically significant with a p value of 0.05.

We examined the effect of EBNA1 expression on DNA repair by comparing FACS profiles of CNE2 and CNE2E after inducing DNA damage with UV or etoposide treatment (Figure 8A). Previous studies have shown that unrepaired DNA damage is reflected by the accumulation of cells in S-phase, while cells that have repaired the damage pass through S and accumulate either in G2/M or G1 depending on which DNA damage checkpoint has been activated [37]–[39]. Hence silencing of PML or a number of DNA repair proteins has been found to increase the percentage of cells in S phase after DNA damage [37],[39]. Similarly, we consistently observed that CNE2E cells had a higher fraction of cells in S phase after UV or etoposide treatment as compared to CNE2 cells (compare profiles ii and v, and profiles iii and vi in Figure 8A) even though the cell cycle distribution of the two cell lines was indistinguishable prior to treatment. In multiple experiments the percentage of CNE2 cells in S phase after UV or etoposide treatment was 55.8±2.5 and 55.4±1.5, respectively, while the same treatments in CNE2E cells resulted in S-phase percentages of 66.5±2.2 and 91.4±0.2, respectively. In both cases, differences with and without EBNA1 are statistically significant with p values <0.001. This effect was confirmed to be due to EBNA1 expression, as down-regulation of EBNA1 in CNE2E with siRNA reduced the S-phase accumulation after DNA damage as compared to the control siRNA treatment against GFP (Figure 8A, compare profiles viii and xi, and profiles ix and xii). Therefore EBNA1 expression results in an impaired ability to repair DNA damage, consistent with the disruption in PML NBs.

The effect of EBNA1 expression in CNE2 cells on apoptosis was also examined by TUNEL assay. The percentage of cells that became TUNEL-positive after etoposide treatment was decreased two-fold in the presence of EBNA1 (Figure 8B), showing that EBNA1 also interferes with apoptosis.

### EBNA1 increases cell survival after DNA damage

We compared the viability of CNE2 and CNE2E cells after etoposide or UV treatment and found that CNE2E cells had a somewhat higher survival rate than CNE2 cells (particularly after etoposide treatment), despite their reduced ability to repair DNA damage (Figure 8C). This is consistent with the known importance of PML NBs in apoptosis [40] and the observed inhibition of apoptosis by EBNA1. The results suggest that EBNA1 promotes the survival of cells even though they contain DNA damage, which has important implications for tumorigenesis.

## Discussion

We have identified a major effect of EBNA1 expression on host cell PML NBs in NPC, where EBNA1 expression results in pronounced loss of PML NBs and the PML protein itself. This effect has important biological implications due to the strong association between PML disruption and tumor development. While initially identified as a gene whose rearrangement leads to promyelocytic leukemia, it has since been found that loss of the PML protein is associated with cancer development for a variety of human tumors [22]. In addition, mice lacking PML develop normally but their cells are more prone to malignant transformation [23].

Unlike the results in NPC cells, we have not seen any notable disruption of PML NBs when EBNA1 is expressed in Hela or 293 cells, suggesting that this effect is specific to particular cell backgrounds. Indeed a previous study examined PML NBs in B-cells latently infected with EBV (both latency and I and latency III forms of infection) and found no obvious difference from uninfected cells [41]. This ability of EBNA1 to disrupt PML NBs in cells of the nasopharnyx could be part of the reason that these cells are particularly susceptible to malignant transformation by EBV.

We found that EBNA1 is partly associated with PML NBs in a native latent infection in NPC cells and can physically associate with at least one PML isoform that appears to be PML IV. We have also shown that disruption of the PML NBs by EBNA1 is due to loss of multiple isoforms of the PML protein and that this effect is proteasome-dependent. This suggests that EBNA1 is targeted to PML bodies through an interaction with PML IV but, once there, can promote the degradation of all PML isoforms.

**Figure 9:**
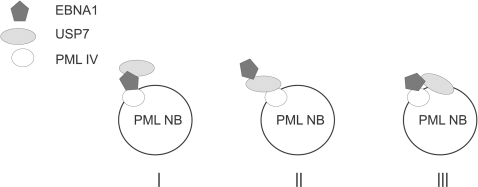
Models of the EBNA1, USP7 and PML interactions. Three possible interpretations of the data on EBNA1-PML and EBNA1-USP7 interactions at PML nuclear bodies are shown.

The disruption of PML NBs by EBNA1 requires EBNA1 binding to USP7, a cellular ubiquitin specific protease that is known to associate with PML NBs [42]. There are several possible scenarios of the role of the EBNA1-USP7 interaction in PML-disruption as depicted in Figure 9. In scenario I, EBNA1 mediates the interaction between a specific PML isoform (ie. PML IV) and USP7 thereby increasing recruitment of USP7 to PML NBs. USP7 could then promote PML degradation either through its catalytic activity or through recruitment of additional cellular proteins. It is not intuitively obvious why deubiquitination would lead to PML destabilization, however approximately three quarters of USP7 is comprised of protein interaction domains, so recruitment of additional cellular enzymes to PML is a viable possibility [15], [17], [43]–[46]. In scenarios II and III, the interaction of EBNA1 with PML NBs depends entirely (scenario II) or partly (scenario III) on USP7. In these cases, loss of PML may require the recruitment of additional cellular proteins by EBNA1, since EBNA1 itself is not known to have any enzymatic activities. For example, we have previously shown that EBNA1 forms a stable complex with casein kinase 2 (CK2) [13] and Scaglioni et al [47] showed that phosphorylation of PML by CK2 targets PML proteins for ubiquitination and subsequent degradation. Therefore it is possible that increased recruitment of CK2 to PML NBs by EBNA1might give the observed effect.

While additional experiments are required to distinguish the above scenarios, the involvement of USP7 in PML disruption by EBNA1 has an interesting parallel with studies of PML disruption by herpes simplex virus type 1 (HSV-1). In HSV-1 infection, ICP0 (also called VMW110) plays a major role in disrupting PML NBs by promoting the degradation of PML protein through its action as an ubiquitin ligase [24],[48]. Although unrelated to EBNA1 in its sequence, ICP0 also binds tightly to USP7 (albeit through a different USP7 domain than EBNA1 [15]) and this interaction is required for PML disruption, at least in part because USP7 stabilizes ICP0 by preventing its autoubiquinitation [29],[49]. While EBNA1 and ICP0 may utilize USP7 in different ways, it is striking that the only two viral proteins known to bind USP7 both require this interaction to facilitate PML disruption.

PML NBs have been shown to be important for p53 activation and DNA repair [36],[39], prompting us to investigate whether the degree of disruption of PML NBs by EBNA1 was sufficient to impair these processes. Indeed both the acetylation of p53 and the repair of DNA lesions after UV or etoposide treatment were found to be impaired by EBNA1. EBNA1-expressing cells, however, survived these treatments as well or better than parental cells, due to an inhibition of apoptosis which also requires PML NBs [35],[50]. The data as a whole support a model in which EBNA1 contributes to the development of NPC through the disruption of PML NBs, thereby increasing the accumulation of DNA damage while promoting cell survival. This model is consistent with reports of frequent but varying chromosomal aberrations in NPC tumors [51],[52]. PML disruption by EBNA1 also provides a mechanistic basis for the observation that EBNA1 expression increases the tumorigenicity of EBV-negative NPC cells [10]. While several viral proteins are known to promote lytic viral infection through disruption of PML NBs, our results indicate that viral proteins can also contribute to carcinogenesis through PML disruption.

## Materials and Methods

### Cell lines

The Saos-2 p53-negative, human osteoblast cell line was cultured in DMEM (Sigma) supplemented with 10% fetal calf serum. The EBV-positive NPC C666-1 cell line and the EBV-negative NPC cell lines HK1 and CNE2Z (CNE2) (both of which lost the EBV genomes after growth in culture) have been previously described [18]–[20]. C666-1 and HK1 were grown in HEPES-modified RPMI 1640 (Sigma), while CNE2 was maintained in alpha minimal essential media (αMEM, Gibco), in all cases supplemented with 10% fetal calf serum. To generate the CNE2E and HK1E cell lines, EBNA1 cDNA was cloned into pcDNA3.1/hygro (−/−; Invitrogen), and CNE2 and HK1 cells were transfected with 10 µg of linearized plasmid. Transfected cells were maintained at low densities in medium supplemented with hygromycin B (Invitrogen; 0.75mg/mL for CNE2 and 0.5mg/mL for HK1) to allow growth of individual colonies. Colonies were examined for EBNA1 expression by IF microscopy, then picked and propagated for further studies in media containing 0.5 mg/mL (CNE2E) and 0.35 mg/mL (HK1E) of hygromycin B, respectively.

### Transfections and RNA interference

To generate CNE2 or Saos-2 cells transiently expressing EBNA1, 1.5×10^5^ cells were transfected with 2 µg of an EBNA1 expressing plasmid containing EBV oriP, pc3OriPE [53], unless otherwise indicated, using lipofectamine 2000 (Invitrogen). Where indicated, the same plasmid expressing EBNA1 mutants Δ325–376 or Δ395–450 was used [13],[54], and the same plasmid lacking EBNA1 cDNA (pc3OriP) was used as a negative control [53]. 48 hrs later, cells were fixed for IF imaging as described below. For RNA interference experiments, 1.5×10^5^ CNE2E or C666-1 cells were transfected with 50 pmol of siRNA against GFP (GCAAGCUGACCCUGAAGUUCAU), against EBNA1 (GGAGGUUCCAACCCGAAAUTT) or against USP7 (UCAAGAUGACUACCAGCUG) using 2 µL of lipofectamine 2000. For C666-1 and one CNE2E sample (Figure 3A and B), cells underwent an identical second round of siRNA transfection 72 hours after the first transfection. For Figure 5C, cells were subjected to three rounds of transfection with siRNA against USP7 or GFP prior to transfection with pc3OriPE.

### Immunofluorescence microscopy

Cells grown on coverslips were fixed with 3.7% formaldehyde in phosphate buffered saline (PBS) for 20 min, rinsed twice in PBS and permeabilized with 1% Triton X-100 in PBS for 5min. Samples were blocked with 4% BSA in PBS followed by incubation with primary antibodies against EBNA1 (R4 rabbit serum at 1∶300 dilution [13]) and PML (Santa Cruz PG-M3 at 1∶50 dilution) and incubation with the secondary antibodies goat anti-rabbit Alexafluor 555 (Molecular Probes) and goat anti-mouse Alexafluor 488 (Molecular Probes) in 4% BSA. Coverslips were mounted onto slides using ProLong Gold antifade medium containing DAPI (Invitrogen). Images were obtained using the 40 x oil objective on a Leica inverted fluorescent microscope and processed using OpenLAB (ver.X.0) software. PML nuclear bodies were quantified by counting all visible PML foci in 100 cells.

### Western blots

Cells were lysed in 9 M urea, 5 mM Tris-HCl (pH 6.8) and briefly sonicated. 100 µg of total protein was subjected to 10% SDS-PAGE and transferred to nitrocellulose. Where indicated, CNE2E cells were treated with 10 µM MG132 for 8 or 10 hours prior to lysis. Membranes were blocked in 5% non-fat dry milk in PBS, then incubated with antibodies against PML (Chemicon AB1370; 1∶2000 dilution), EBNA1 (OT1X at 1∶2000; kindly supplied by Japp Middeldorp), actin (Ab-1, Oncogene Research Products; 1∶20 000), Sp100 (Chemicon 1380, 1∶1000 dilution) or USP7 (rabbit serum against full-length USP7). After washing, blots were probed with goat anti-mouse peroxidase (1∶3000) or goat anti-rabbit peroxidase (1∶5000) from Santa Cruz, then developed using chemiluminescence reagents (ECL, Perkin Elmer). Membranes were stripped in 0.1 M glycine pH 2.9 for 30 min, washed in PBS-Tween, blocked and re-probed with the next antibody as described above.

Experiments in Figure 7 were performed as above except that some cells were treated with 10 µg/mL of etoposide 24 hrs prior to harvesting and 80 µg (p53 blot) or 60 µg (acetyl-p53 blot) of total protein was analyzed by Western blotting using the following antibodies: PAb 1801 for p53 [55] (a gift from Sam Benchimol) and antibody 2525 for acetyl-p53 K382 (Cell Signaling Technologies). For acetyl-p53 blots, membranes were blocked and incubated with primary antibody in 5% BSA, 50 mM Tris pH7.4, 150 mM NaCl, 0.1% Tween-20. The same experiment was also performed in Hela cells transfected with pc3OriP or pc3OriPE. In the later case, EBNA1 expression was confirmed in approximately 80% of the cells by IF prior to etoposide treatment (10 µg/mL) 48 hours post-transfection. 100 µg of cell lysate was Western blotted as described above except that the anti-p53 antibody was DO-1 from Santa Cruz (sc-126).

### Co-immunoprecipitation

Log phase C666-1 cells were lysed in IP buffer (20 mM Tris-HCl pH 7.5, 150 mM NaCl, 1 mM MgCl_2_, 10% glycerol, 1% Triton X-100, and protease inhibitors) on ice for 30 min. After centrifugation, the supernatant was pre-cleared with protein A/G agarose (Santa Cruz) and then equal amounts (4 mgs) were incubated for 4 hr at 4°C with either mouse IgG coupled to agarose (Santa Cruz; negative control) or with OT1X EBNA1 monoclonal antibody followed by protein A/G agarose overnight at 4°C. Beads were collected by centrifugation, washed in IP buffer then boiled in SDS loading buffer. Immunoprecipitated proteins were separated by SDS-PAGE and Western blotted as described above. The positions of PML I and PML IV isoforms on Western blots was determined by transfecting C666-1 cells with constructs expressing FLAG-PML I or FLAG-PML IV [56] and analyzing 30 µg of lysate by Western blotting using anti-FLAG antibody (Abcam AB21536; 1∶10,000 dilution).

### FACS analysis

CNE2 and CNE2E cells (before and after siRNA treatment) seeded at 60% confluence in 10 cm dishes were treated with UV (50×10^2^ µJ/cm^2^) or 10 µg/mL of etoposide. 24 hours later, adherent cells were harvested, fixed in 70% ethanol, treated with RNAse (50 µg/mL) at 37^o^C for 1 hour and stained with propidium iodide. DNA content was analyzed immediately after propidium iodide treatment on a FACScalibur (Becton Dickinson, USA) and cell cycle analysis was performed using Modfit LT 3.1 (Verity Software House).

### Apoptosis Assay

CNE2 and CNE2E cells were treated with etoposide as stated above. 48 hours later, cells were processed for TUNEL staining using the APO-BrdU TUNEL Assay Kit (Invitrogen, MP23210) according to the manufacturer's instructions. Cells were then mounted on coverslips, counter-stained with DAPI and analyzed by fluorescence microscopy. Apoptotic index was calculated as the number of TUNEL-positive cells divided by the total number of cells. The experiment was done in triplicate and at least 100 cells were counted for each sample.

### Cell viability assay

Cell viability was measured using Trypan blue (Gibco) exclusion assay as follows: CNE2 and CNE2E cells were seeded in 12-well plates such that they reached 80–90% confluence at time of harvesting. 24 hours later, cells were treated with etoposide (10 µg/mL) or UV (50×10^2^ µJ/cm^2^). After growing for 24, 48 and 72 hours, floating and adherent cells were harvested, washed in PBS, stained with 0.04% Trypan blue in PBS and counted immediately. Experiments were done in triplicate and at least 200 cells were counted for each replicate.

## Supporting Information

Figure S1 Quantification of EBNA1 levels in NPC cell lines. (A) C666-1, HK1E, CNE2E and CNE2 cells were lysed in 9 M urea, 10mM Tris pH6.8 and 100 µg of protein from each sample was analysed by Western blotting using antibodies against EBNA1 and actin and the ECL Plus system (Perkin Elmer). Positions of the full length EBNA1 (EBNA FL) expressed endogenously in C666-1 and recombinant EBNA1 lacking most of the Gly-Ala repeat region (EBNA1ΔGA) expressed in HK1E and CNE2E are indicated. (B) Flourescent signals from each band were quantified on a Typhoon Imaging scanner using ImageQuant 5.0 software. EBNA1 levels were normalized to the actin loading control and are shown relative to the C666-1 value.
(0.20 MB TIF)

Figure S2 Quantification of PML mRNA levels in NPC cell lines. Total RNA from C666-1, CNE2E and CNE2 cells was harvested using RNeasy mini kit (Qiagen). RNA quality was assessed by confirmation of intact 28S and 18S ribosomal bands following agarose gel electrophoresis and ethidium bromide staining. cDNA was synthesized using 1 µg total RNA and First Strand cDNA sythesis kit (Fermentas). Quantitative real-time PCR was performed with 1/5 to 1/20 of the cDNA template and Platinum SYBR Green qPCR SuperMix-UDG (Invitrogen) in a Rotorgene qPCR System (Corbett Research). The primer pairs used to amplify PML mRNA were CGGAGGAGGAGTTCCAGTTT and CCACAATCTGCCGGTACAC. β-actin mRNA was amplified from the same samples using the primers CATGTACGTTGCTATCCAGGC and CTCCTTAATGTCACGCACGAT. PML mRNA levels are shown realtive to β-actin mRNA.
(0.07 MB TIF)

Figure S3 Interactions of EBNA1 with overexpressed FLAG-tagged PML I and PML IV. CNE2 cells were co-transfected with pc3oriPE (expressing EBNA1) and plasmids expressing either FLAG-PML I, FLAG-PML IV or FLAG-tagged lacZ (negative control). 16 hours later, IPs were performed using anti-FLAG M2 agarose beads. Recovered proteins were immunoblotted for FLAG and EBNA1. Input samples contain 1/50th the amount of lysate used in the FLAG IPs.
(0.18 MB TIF)
