# Supplementary figures and images for: Epstein-Barr Nuclear Antigen 1 Contributes to Nasopharyngeal Carcinoma through Disruption of PML Nuclear Bodies
Source: PLoS Pathog. 2008 Oct 3;4(10):e1000170. doi: 10.1371/journal.ppat.1000170 (PMC2542412; doi:10.1371/journal.ppat.1000170)

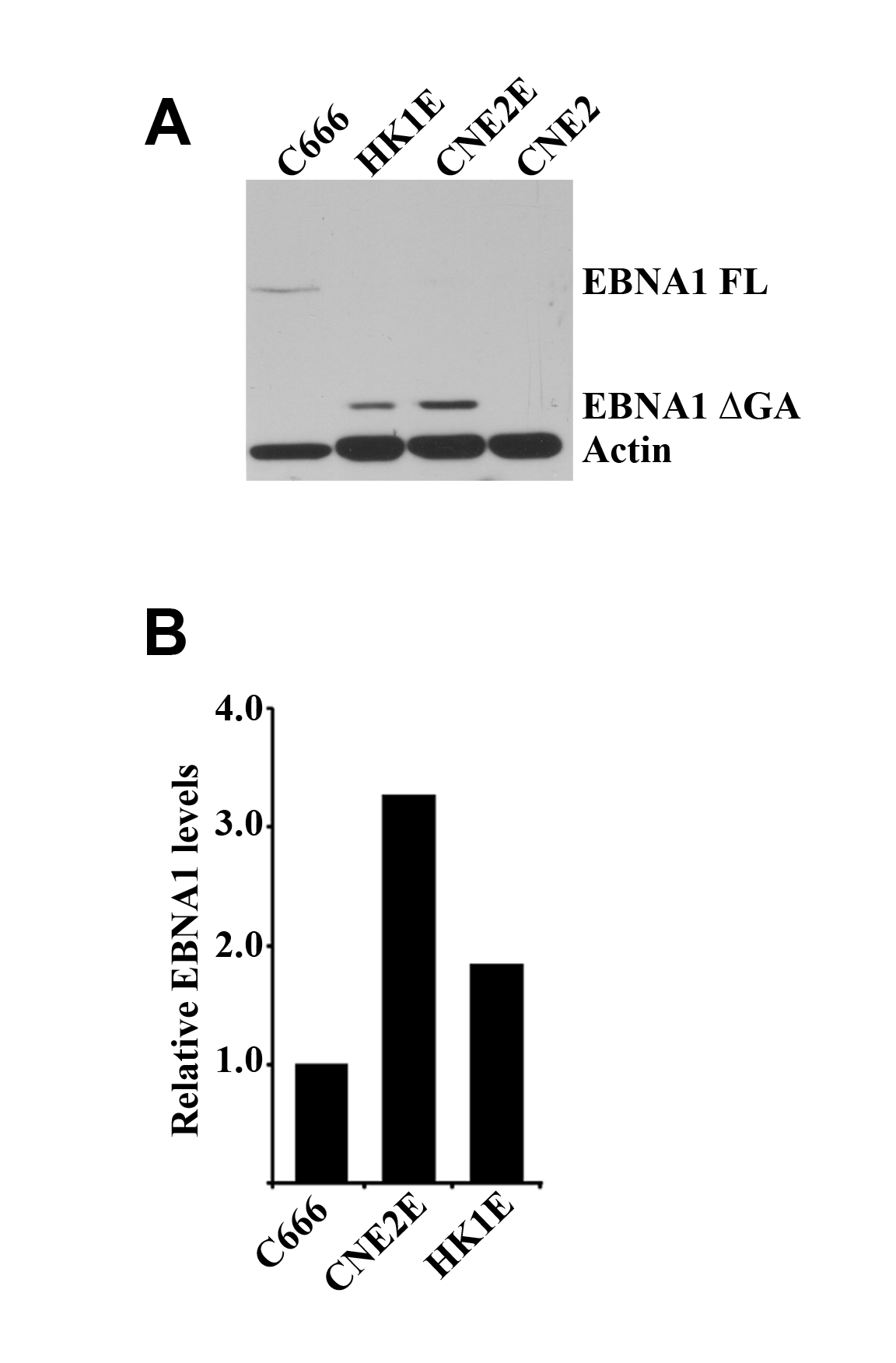

Supplement: Figure S1 — Quantification of EBNA1 levels in NPC cell lines. (A) C666-1, HK1E, CNE2E and CNE2 cells were lysed in 9 M urea, 10mM Tris pH6.8 and 100 µg of protein from each sample was analysed by Western blotting using antibodies against EBNA1 and actin and the ECL Plus system (Perkin Elmer). Positions of the full length EBNA1 (EBNA FL) expressed endogenously in C666-1 and recombinant EBNA1 lacking most of the Gly-Ala repeat region (EBNA1ΔGA) expressed in HK1E and CNE2E are indicated. (B) Flourescent signals from each band were quantified on a Typhoon Imaging scanner using ImageQuant 5.0 software. EBNA1 levels were normalized to the actin loading control and are shown relative to the C666-1 value. (0.20 MB TIF) [file ppat.1000170.s001.tif]

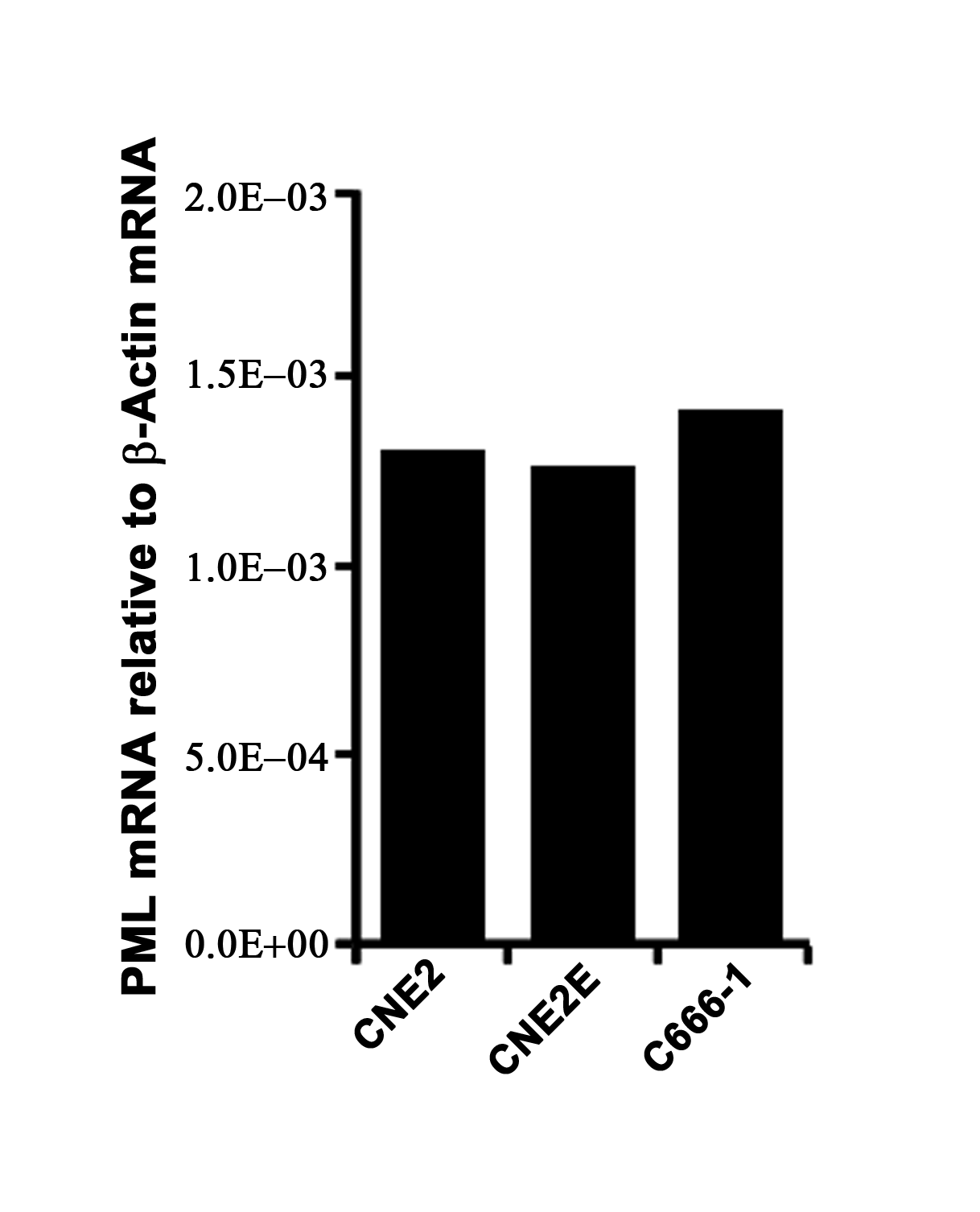

Supplement: Figure S2 — Quantification of PML mRNA levels in NPC cell lines. Total RNA from C666-1, CNE2E and CNE2 cells was harvested using RNeasy mini kit (Qiagen). RNA quality was assessed by confirmation of intact 28S and 18S ribosomal bands following agarose gel electrophoresis and ethidium bromide staining. cDNA was synthesized using 1 µg total RNA and First Strand cDNA sythesis kit (Fermentas). Quantitative real-time PCR was performed with 1/5 to 1/20 of the cDNA template and Platinum SYBR Green qPCR SuperMix-UDG (Invitrogen) in a Rotorgene qPCR System (Corbett Research). The primer pairs used to amplify PML mRNA were CGGAGGAGGAGTTCCAGTTT and CCACAATCTGCCGGTACAC. β-actin mRNA was amplified from the same samples using the primers CATGTACGTTGCTATCCAGGC and CTCCTTAATGTCACGCACGAT. PML mRNA levels are shown realtive to β-actin mRNA. (0.07 MB TIF) [file ppat.1000170.s002.tif]

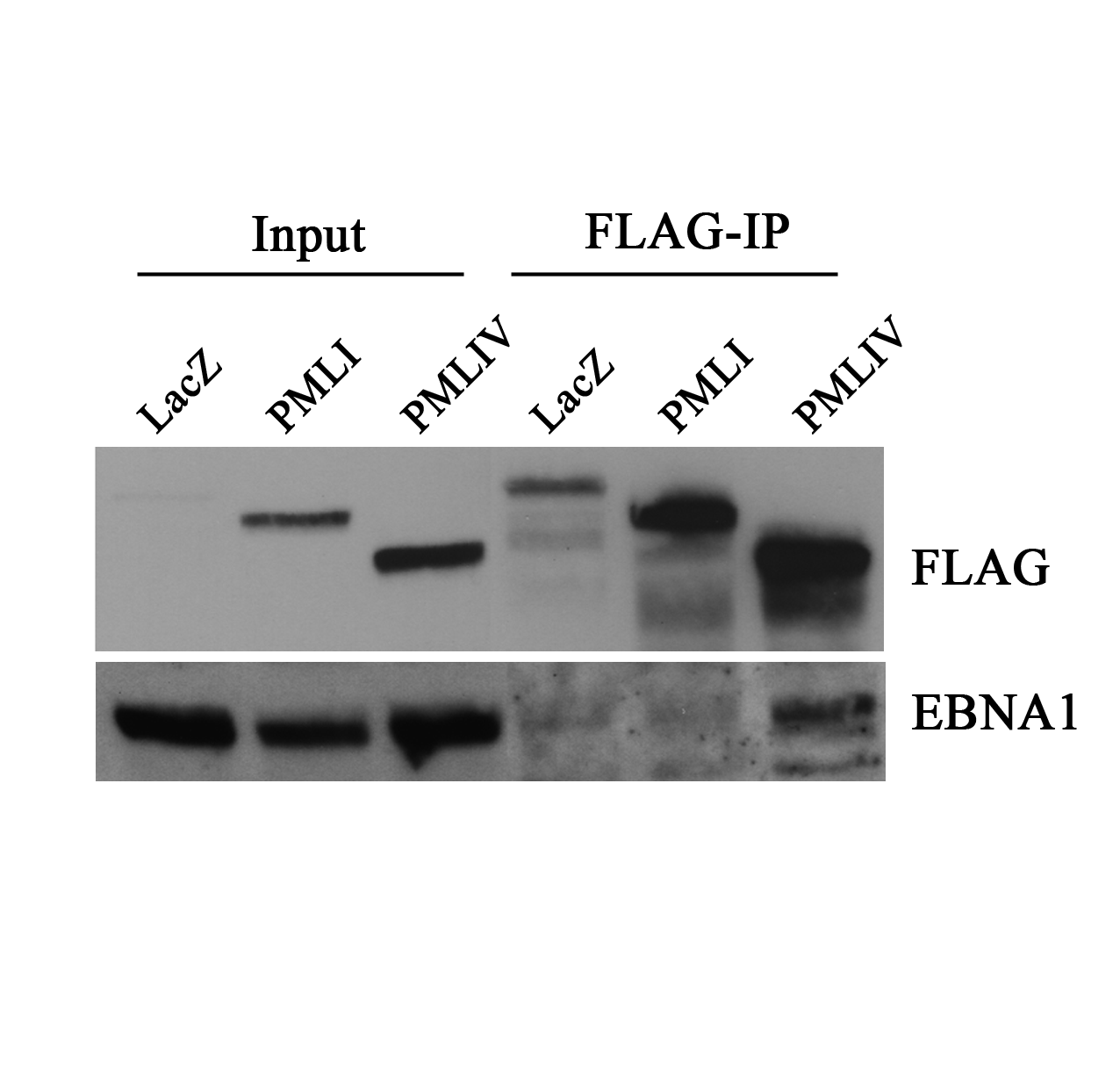

Supplement: Figure S3 — Interactions of EBNA1 with overexpressed FLAG-tagged PML I and PML IV. CNE2 cells were co-transfected with pc3oriPE (expressing EBNA1) and plasmids expressing either FLAG-PML I, FLAG-PML IV or FLAG-tagged lacZ (negative control). 16 hours later, IPs were performed using anti-FLAG M2 agarose beads. Recovered proteins were immunoblotted for FLAG and EBNA1. Input samples contain 1/50th the amount of lysate used in the FLAG IPs. (0.18 MB TIF) [file ppat.1000170.s003.tif]
